# Transcriptional Dysregulation of Upstream Signaling of IFN Pathway in Chronic HCV Type 4 Induced Liver Fibrosis

**DOI:** 10.1371/journal.pone.0154512

**Published:** 2016-05-02

**Authors:** Marwa K. Ibrahim, Ghada Maher Salum, Noha G. Bader El Din, Reham M. Dawood, Ahmed Barakat, Ahmed Khairy, Mostafa K. El Awady

**Affiliations:** 1 Department of Microbial Biotechnology, Genetic Engineering Division, National Research Centre, Dokki, Giza, Egypt; 2 Department of Microbiology, Faculty of Science, Ain Shams University, Cairo, Egypt; 3 Endemic Medicine Department, Faculty of Medicine, Cairo University, Giza, Egypt; University of Sydney, AUSTRALIA

## Abstract

IFN orchestrates the expression of various genes to halt hepatitis C virus (HCV) replication with the possibility of either reduced or increased liver fibrosis; due to controlled viral replication or overproduction of inflammatory mediators, repectively. In this study, we examined the transcriptional profiling of type I IFN related genes in HCV-chronically infected patients with varying degrees of liver fibrosis. PCR array was used to examine the expression of 84 type I IFN related genes in peripheral blood mononuclear cells (PBMCs) RNA from 12 treatment-naïve chronic HCV patients (5 F0-F1 and 7 F2-F4) and 5 healthy subjects. We further validated our results by quantitative real time PCR (qRT-PCR) in 103 treatment-naïve chronic HCV patients (43 F0-F1 and 60 F2-F4) and 15 controls. PCR array data revealed dysregulation in TLR7 pathway. The expression of TLR7 was decreased by 4 folds and MyD88 was increased by 3 folds in PBMCs of F2-F4 patients when compared to the healthy volunteers (p = 0.03 and 0.002, respectively). In addition, IRF7 and TLR7 showed dramatic downregulation (6 and 8 folds, respectively) in F2-F4 patients when compared to F0-F1 ones. qRT-PCR confirmed the altered expression patterns of TLR7 and MyD88 in F2-F4 patients when compared to either controls or F0-F1 patients. However, by qRT-PCR, IRF7 and NF-κB1 (TLR7 pathway transcription factors) exhibited similar mRNA abundance among F2-F4 and F0-F1 patients. These results suggest that TLR7 and MyD88 are possible candidates as biomarkers for the progression of HCV-induced liver fibrosis and/ or targets for therapeutic intervention.

## Introduction

Hepatic fibrosis is a progressive disease resulting from excessive wound healing process associated with chronic liver injury [1]. Liver fibrosis develops from several etiologies such as viral hepatitis (hepatitis C and B), alcoholic liver disease, and non-alcoholic fatty liver disease with hepatitis C virus (HCV) being the leading cause of liver fibrosis [2]. HCV prevalence covers around 3% of the world’s population [3]. Although the therapeutic intervention of HCV has recently been improved as the consequence of discovering direct acting antiviral agents (DAAs), the majority of patients are still at risk of fibrosis progression that is culminating in cirrhosis in 20–30% and hepatocellular carcinoma (HCC) in 4% of the cases [4].

Type I interferon (IFN) plays a crucial role in combating HCV. Following HCV sensing by host cell, type I IFN is ultimately induced by several innate pathways including Toll like receptor (TLR) 2, 3, 7, and 8 [5]. Engagement of HCV viral single-stranded RNA (ssRNA) by TLR7 triggers downstream signaling staring with the recruitment of the adaptor molecule myeloid differentiation primary response gene 88 (MyD88) and ending with the activation of transcription factors IRF7 and NF-κB1, which induce the expression of type I IFN and inflammatory cytokines and chemokines, respectively [5]. Upon binding to its cognate receptor, type I IFN induces IFN-stimulated genes (ISGs) that induce cell apoptosis leading to the elimination of virus-infected cells [6].

Multiple studies have linked genetic variants and expression pattern of type I IFN related genes to the susceptibility to HCV infection and response to IFN therapy. Dysregulation of ISGs in liver biopsy was shown to be a predictor of response to IFN therapy in HCV infection [7]. Microarray analysis demonstrated up-regulation of many genes associated with type I IFN pathway in liver biopsies [8] and peripheral blood mononuclear cells (PBMCs) [9] from HCV-chronically infected patients compared to healthy subjects. TLRs7/8 showed differential expression in monocytes of chronic HCV patients shown to be responders to IFN treatment [10]. The expression levels of type I IFN related genes in PBMCs and their correlation to the severity of liver fibrosis in HCV genotype 4 infected patients are not well addressed. We showed previously association between single nucleotide polymorphism in ISGs (OAS and MyxA) and response to IFN therapy as well as progression of hepatic fibrosis in Egyptian patients infected with HCV genotype 4 [11, 12]. In the present study we investigated the transcriptional profiling of several genes implicated in type I IFN in PBMCs derived from genotype 4 chronically infected patients having various grades of hepatic fibrosis using pathway focused PCR array. Abundance of mRNA of several type I IFN pathway mediated genes was altered in HCV patients with advanced fibrosis compared to either healthy controls or patients with early fibrosis. We have then validated the noticeable dysregulation of a couple of key signaling molecules, TLR7 and MyD88 besides the transcription factors NF-κB1 and IRF7 in a larger cohort of patients by quantitative real time PCR (qRT-PCR).

## Methods

### Ethical statement

All experiments were approved by the institution ethical review board (medical research ethics committee at National Research Center, Cairo, Egypt) according to Helsinki Declaration 1975 revised in 2008 and performed with the understanding and the consent of the human subject. Written informed consent was obtained from each subject before collecting blood samples and the consent procedure was approved by ethics committee/institutional review board.

### HCV-chronically infected patients

This study included 103 treatment-naïve patients with chronic genotype 4 HCV infection recruited from Kasr Al-Aini Viral Hepatitis Center, Faculty of Medicine, Cairo University and Viral Hepatitis Center, Ahmed Maher Teaching Hospital. The selected patients were HCV seropositive and having detectable serum HCV-RNA. None of the patients had HBV surface antigen (HBsAg), anti-schistosoma antibodies, autoimmune markers, uncontrolled type II diabetes mellitus, history of drug abuse and alcohol consumption or any other etiologies implicated in chronic liver diseases. The severity of liver fibrosis was determined histologically in liver biopsies by Knodell and Metavir scoring system and confirmed by transient elastography (fibroscan) measurement. Chronic HCV patients were categorized into 2 groups based on the degree of liver fibrosis; early fibrosis (F0-F1, n = 43) and late fibrosis (F2-F4, n = 60).

### Healthy subjects

The selected 15 healthy subjects had no history of HCV infection (negative for IgG anti-HCV Abs and serum HCV-RNA), and were negative for HBsAg, anti-schistosoma antibodies, autoimmune antibodies as well as had normal liver enzymes and with no overt liver diseases.

### RNA extraction

Total cellular RNA was extracted from the freshly collected blood using the single-step method [13]. RNA samples were quantified using a Thermo Scientific NanoDrop™ Spectrophotometer and kept at -80°C.

### PCR Array and qRT-PCR

Total cellular RNA (800 ng for PCR array experiments and 250 ng for individual primer based qRT-PCR experiments) was reverse transcribed into cDNA using RT^2^ PCR First Strand Kit (SABiosciences, Valencia, CA). For human type I IFN response RT^2^ Profiler™ PCR array experiments (SABiosciences), 102 μl of the synthesized cDNA was mixed with 1150 μl RT^2^ SYBR Green/ROX qPCR master mix (SABiosciences) in a final volume of 2300 μl. In qRT-PCR, 1 μl of cDNA and 1 μl of gene-specific PCR primer for human IRF7, NF-κB1, TLR7, or MyD88 (10 μM; SABiosciences) were mixed together with RT^2^ SYBR Green/ROX qPCR master mix (SABiosciences) in a final volume of 25μl. The house keeping gene human B2M (SABiosciences) was used in a separate reaction as endogenous reference in order to normalize differences in the amount of input cDNA in each assay. The thermal profile followed was; intial incubation for 10 min at 95°C (AmpliTaq Gold pre-activation), followed by 40 cycles at 95°C for 15 sec and 60°C for 1 min. The PCR run was performed on Rotor Gene real-time PCR system (Qiagen, Santa Clarita, CA). Relative expression of each gene was calculated by the 2^-ΔΔCT^ method, and differences in gene expression were compared to the mean of the healthy group and expressed as fold-change.

### Genomic DNA extraction and genotyping of IL-28B rs12979860 polymorphism

DNA was extracted from peripheral blood using Qiagen DNA extraction kit (Qiagen), following the manufacturer’s instructions. IL-28B rs12979860 polymorphism was analysed by polymerase chain reaction as mentioned previously [14] which is followed by restriction fragment length polymorphism (PCR-RFLP); the 139 bp amplified PCR products was digested overnight at 37°C using 1 unit of the BstU-I restriction endonuclease (New England Biolabs, Hitchin, UK). The digested products were analyzed on 4% agarose gel. Getting the uncut fragment of 139 bp only refered to the homozygous TT genotype, 109 bp only refered to the homozygous CC genotype and the two fragments of 139 and 109 bp refered to the heterozygous CT genotype.

### ELISA

The level of circulating MyD88 was measured in serum samples using enzyme-linked immunosorbent assay (ELISA) kit (R&D systems, Minneapolis, MN, USA) following the manufacturer’s instructions. The range of detection limit by ELISA was 0.313–20 ng/mL.

### Pathway-enrichment analysis

WebGestalt [15] was used to cluster the genes sharing common pathway using Wikipathway analysis with a hypergeometric distribution algorithm and cut off at P < 0.01.

### Statistical analysis

The data were analyzed using Prism software (GraphPad, La Jolla, CA) and either the parametric unpaired t test or the non-parametric Mann-Whitney U test was used depending on the normal distribution of the data. Data were presented as the mean and standard error of the mean unless mentioned. Difference between groups was considered statistically significant if p≤ 0.05.

## Results

### Clinical data of HCV patients

The demographic data of the studied patients are summarized in Tables 1 and 2. The two groups of the 12 HCV infected patients (F0-F1 *vs* F2-F4) selected for the PCR array experiment did not show any significant differences in gender, body mass index (BMI), age, ALT, AST, platelets count and prothrombin whereas patients with late fibrosis (F2-F4) had lower level of albumin (Table 1). When the comparison was made between the two groups of 103 HCV-chronically infected patients selected for the validation qRT-PCR experiments, patients with late fibrosis (F2-F4) showed significant differences in platelets count and prothrombin when compared to patients with early fibrosis (F0-F1). Other demographic parameters including gender, BMI, age, albumin, ALT and AST did not reveal any significant differences between the two patient groups (Table 2).

**Table 1:** Clinical features of chronic HCV infected patients with early and late liver fibrosis selected for the PCR array experiment.

	Early fibrosis (F0-F1) n = 5	Late fibrosis (F2-F4) n = 7	*P*
**Female/ Male**	0/5	0/7	ns
^1^**Age (years)**	36 ± 5.1	46.3 ± 4.7	ns
**BMI (kg/m2)**	24.45 (20–33.2)	23.7 (22.4–27.7)	ns
^1^**Albumin (g/dL)**	4.4 ± 0.2	3.6 ± 0.2	0.04
^1^**AST (U/L)**	23.5 ± 2.7	33.2 ± 6.6	ns
^1^**ALT (U/L)**	30.3 ± 6.5	32.3 ± 9.8	ns
**Platelets count (cmm3)**	257.5 (150–320)	210 (80–286)	ns
**Prothrombin (%)**	99 (80–100)	82 (65–92)	ns

^1^ Data are expressed as mean and standard error of mean.

“n” refers to the sample size.

BMI, platelets, and prothrombin data are expressed as median and range.

**Table 2:** Clinical features of chronic HCV infected patients with early and late liver fibrosis selected for the validation qRT-PCR experiments.

	Early fibrosis (F0-F1) n = 43	Late fibrosis (F2-F4) n = 60	*P*
**Female/ Male**	20/23	32/28	ns
^1^**Age (years)**	38 ± 2.2	43± 2	ns
^1^**BMI (kg/m2)**	27.5 ± 0.6	27±3.3	ns
^1^**Albumin (g/dL)**	4.2 ± 0.1	4.1 ± 0.07	ns
^1^**AST (U/L)**	26.6 ± 3.3	36.5 ± 4.6	ns
^1^**ALT (U/L)**	27.7± 3.6	36.9 ± 4.6	ns
**Platelets count (cmm3)**	303.0 (181–370)	250.0 (80–420)	0.05
**Prothrombin (%)**	96 (80–100)	90 (70–100)	0.04

^1^ Data are expressed as mean and standard error of mean.

“n” refers to the sample size.

Platelets and prothrombin data are expressed as median and range.

### Hepatic fibrosis is associated with altered mRNA expression of type I IFN pathway related genes in PBMCs of HCV-chronically infected patients.

The influence of chronic HCV infection on hematological gene expression remains unclear in particular from the aspect of disease progression. Therefore, we investigated the impact of HCV-induced hepatic fibrosis on blood transcriptional profile of key genes involved in type I IFN pathway; the fundamental arm of innate immunity against HCV infection. Pathway focused PCR array was used to analyze the gene expression of panel of 84 mediators of type I IFN pathway in PBMCs RNA from 12 treatment-naïve chronic HCV patients (5 F0-F1 and 7 F2-F4) and 5 healthy subjects (see Table in S1 Table for the full data set). Out of 84 genes only 14 genes fulfilled the criteria of showing ≥ two folds differential regulation plus statistical significance at p≤ 0.05 upon comparing either F0-F1 or F2-F4 to healthy subjects (see Table 3). Overall, all differentially regulated genes showed a trend of up regulation in HCV chronically infected patients with early or late fibrosis except for TLR7 (Table 3). Several transcripts showed upregulation in early fibrosis group (F0-F1) compared with controls and returned to the basal levels as the disease progressed i.e in F2-F4 (EIF2AK2, IFITM2, JAK1, JAK2, STAT3, and TICAM1 (Table 3, p≤ 0.05 for all)), and these are likely involved in the early immune defense against HCV infection. We observed upregulation of several transcripts in late fibrotic patients as compared to the controls (MyD88, CD70, CRP, IFNE, IL6, IRF3 and NOS2 (Table 3, p< 0.05 for all)) and down regulation of a sole transcript (TLR7) (Table 3, p = 0.03). When the comparison was made between early and late fibrosis groups, 12 genes showed significant downregulation in F2-F4 compared to F0-F1 patients (more than two folds differential regulation associated with p≤ 0.05 (Table 4)). The 12 differentially regulated genes included mediators in IFN downstream signaling pathway (IFNAR1, STAT2, STAT3, and JAK1), ISGs (IFI6, IFIT2, and MX2), and mediators in IFN upstream signaling pathway (TICAM1, TLR7, TLR8, and IRF7) as well as the cytokine CCL2 (Table 4). To investigate if the altered expression of ISGs (IFI6, IFIT2, and MX2) is driven by type III IFN we did genotyping of rs12979860 (the most prominent SNP in IL28B (located 3 kb upstream of IL-28B)) on DNA isolated from PBMCs of the tweleve studied patients. We found similar expression of the three studied ISGs among CC genotype (major allele) bearing patients and CA/AA (minor allele) bearing patients (Figure in S1 Fig). By using pathway-enrichment analysis (WebGestalt) for all differentially regulated genes in the networks, it was particularly interesting to find dysregulation in TLR7 cascade pathway. The expression of the pathway receptor (TLR7) was decreased by 4 folds in PBMCs of patients with late fibrosis while the expression of the pathway adaptor molecule (MyD88) was increased by 3 folds in the same patients when compared to the healthy volunteers (Table 3; p = 0.03 and 0.002, respectively). Moreover, The expression of TLR7 and the pathway transcription factor (IRF7) was decreased by 8 and 5 folds respectively in PBMCs of patients with late fibrosis when compared to those with early fibrosis (Table 4; p = 0.009 for TLR7 and 0.04 for IRF7). Collectively, these data indicated that HCV infection modulated TLR7 signaling pathway in PBMCs. These differences were most evident after the progression of liver fibrosis i.e. F2-F4 vs C or F0-F1.

**Table 3:** Differential expression of 14 type I IFN pathway genes showing statistically significant regulation in HCV-chronic infected patients compared with healthy controls.

**Gene Symbol**	**Gene description**	**Unigene**	**Refseq**	**Fold regulation F0-F1/ C**	***P* value**
JAK2	Janus kinase 2	Hs.656213	NM 004972	4.7502	0.003
STAT3	Signal transducer and activator of transcription 3	Hs.463059	NM 003150	3.5259	0.007
JAK1	Janus kinase 1	Hs.207538	NM 002227	2.385	0.01
TICAM1	Toll-like receptor adaptor molecule 1	Hs.29344	NM 182919	2.2815	0.01
EIF2AK2	Eukaryotic translation initiation factor 2-alpha kinase 2	Hs.131431	NM 002759	3.0022	0.04
IFITM2	Interferon induced transmembrane protein 2 (1-8D)	Hs.709321	NM 006435	3.6859	0.04
**Gene Symbol**	**Gene description**	**Unigene**	**Refseq**	**Fold regulation F2-F4/ C**	***P* value**
MyD88	Myeloid differentiation primary response gene (88)	Hs.82116	NM 002468	2.6987	0.002
IFNE	Interferon, epsilon	Hs.682604	NM 176891	17.9796	0.02
CRP	C-reactive protein, pentraxin-related	Hs.76452	NM 000567	25.6647	0.02
NOS2	Nitric oxide synthase 2, inducible	Hs.709191	NM 000625	20.2722	0.03
TLR7	Toll-like receptor 7	Hs.659215	NM 016562	-3.8151	0.03
IL6	Interleukin 6 (interferon, beta 2)	Hs.654458	NM 000600	4.754	0.04
IRF3	Interferon regulatory factor 3	Hs.289052	NM 001571	3.0902	0.04
CD70	CD70 molecule	Hs.715224	NM 001252	4.7016	0.04

Relative expression of 14 most regulated IFN pathway genes (p ≤0.05 and at least twice up/or down regulation) in F0-F1 or F2-F4 patients compared to healthy controls (C).

A negative sign indicates reduced expression in the patients relative to the normal ones.

Genes are arranged in a descending order based on their significant p value.

**Table 4:** Differential expression of type I IFN pathway genes showing statistically significant regulation in HCV-chronic infected patients with late liver fibrosis compared to patients with early liver fibrosis.

Gene Symbol	Gene description	Unigene	Refseq	Fold regulation F2-F4/ F0-F1	*P* value
JAK1	Janus kinase 1	Hs.207538	NM 002227	-28.3867	0.001
IFIT2	Interferon-induced protein with tetratricopeptide repeats 2	Hs.437609	NM 001547	-61.4294	0.008
TLR7	Toll-like receptor 7	Hs.659215	NM 016562	-7.7797	0.009
STAT2	Signal transducer and activator of transcription 2	Hs.530595	NM 005419	-6.6359	0.01
IFNAR1	Interferon (alpha, beta and omega) receptor 1	Hs.529400	NM 000629	-10.8035	0.02
IFI6	Interferon, alpha-inducible protein 6	Hs.523847	NM 002038	-3.7034	0.02
MX2	Myxovirus (influenza virus) resistance 2	Hs.926	NM 002463	-21.1289	0.03
CCL2	Chemokine (C-C motif) ligand 2	Hs.303649	NM 002982	-3.2671	0.03
TICAM1	Toll-like receptor adaptor molecule 1	Hs.29344	NM 182919	-2.1406	0.03
IRF7	Interferon regulatory factor 7	Hs.166120	NM 001572	-5.5426	0.04
STAT3	Signal transducer and activator of transcription 3	Hs.463059	NM 003150	-23.6913	0.05
TLR8	Toll-like receptor 8	Hs.660543	NM 138636	-2.5811	0.05

Relative expression of 12 most regulated IFN pathway genes (p ≤0.05 and at least twice up/or down regulation) in late fibrosis patients (F2-F4) compared to early fibrosis (F0-F1).

A negative sign indicates reduced expression in F2-F4 patients relative to the F0-F1 ones.

Genes are arranged in a descending order based on their significant p value.

### HCV-induced hepatic fibrosis is associated with dysregulated TLR7 cascade pathway

**Fig 1:**
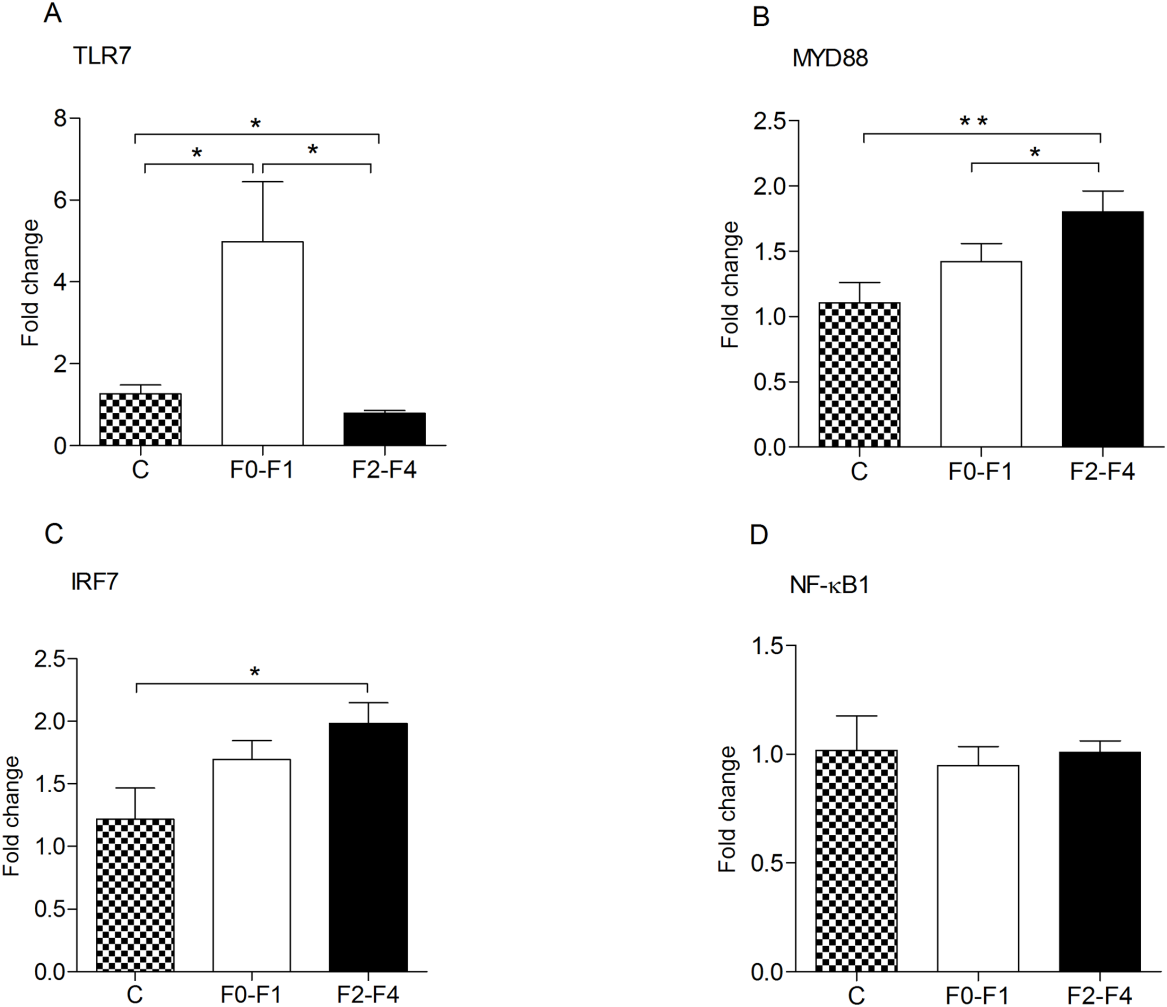
Diagrammatic representation of the expression pattern of TLR7 pathway key related transcripts in early versus late fibrotic patients. qRT-PCR was used to quantify the mRNA expression of TLR7 (A), MyD88 (B), IRF7 (C) and NF-κB1 (D) in PBMCs of healthy controls (C, n = 15) and HCV infected patients with early hepatic fibrosis (F0-F1, n = 43) and with late hepatic fibrosis (F2-F4, n = 60). The data were normalized to the expression of B2M and are shown as the fold-change relative to the mean of healthy subjects. (*, p<0.05; **, p<0.01).

To further confirm the remarkable dysregulation of TLR7 pathway, we examined the expression pattern of TLR7 pathway mediators (TLR7, MyD88, IRF7 and NF-κB1) using qRT-PCR assay on large case numbers (15 healthy subjects and 103 HCV-infected patients). The same expression pattern of TLR7 and MyD88 genes in PBMCs of fibrotic patients as obtained by PCR array were confirmed by qRT-PCR (Fig 1A and 1B). TLR7 showed dramatic increase in its expression (5 folds, p = 0.01) in F0-F1 group compared to healthy subjects (Fig 1A), and then tredemenous decrease in its expression in F2-F4 group when compared either to healthy subjects or F0-F1 groups (Fig 1A, p = 0.04 and 0.02, respectively). The late fibrosis group (F2-F4) showed significant up regulation of MyD88 transcript when compared to either healthy subjects or early fibrosis group (F0-F1) (Fig 1B, p = 0.009 and 0.03, respectively). In contrast to PCR array data, IRF7 showed significant upregulation in late fibrosis group as compared to healthy controls (Fig 1C, p = 0.04), however, differential expression of IRF7 transcript between early and late fibrosis groups was not confirmed by qRT-PCR (Fig 1C). We sought to investigate the expression of NF-κB1(the other TLR7 pathway transcription factor that was not included in the PCR array). Similarily, NF-κB1 mRNA abundance showed no significant changes in regulation throughout different stages of fibrosis (Fig 1D). Together, the altered expression of TLR7 and MyD88 in patients with advanced fibrosis confirms the dysregulation of TLR7 signaling pathway observed from the PCR array data.

### HCV-induced hepatic fibrosis is associated with elevated serum level of MyD88 protein

**Fig 2:**
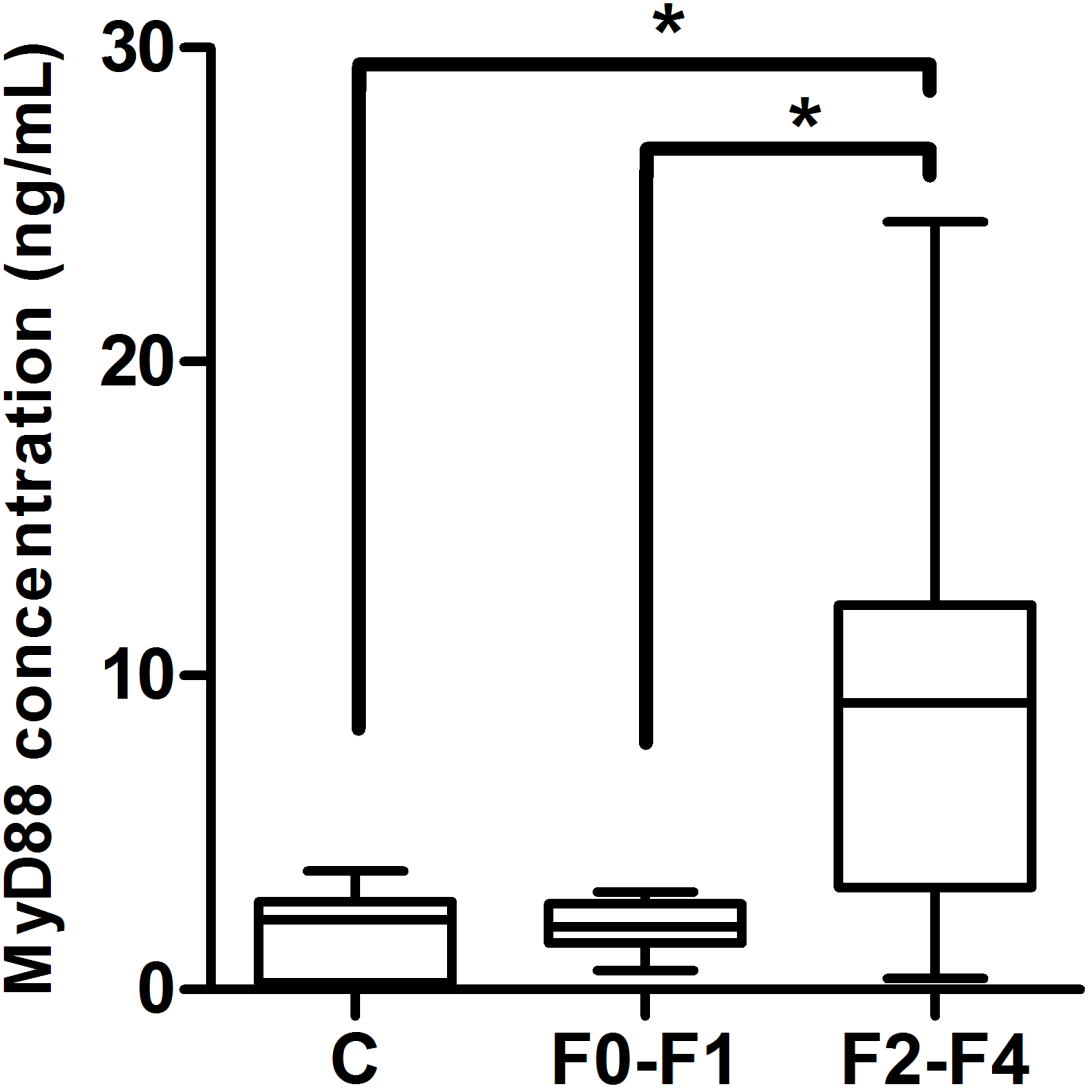
Diagrammatic representation of the serum level of MyD88 protein in early versus late fibrotic patients. ELISA was used to measue the protein level of secreted MyD88 in serum from healthy controls (C, n = 15) and HCV infected patients with early hepatic fibrosis (F0-F1, n = 43) and with late hepatic fibrosis (F2-F4, n = 60). Statistical comparison was performed using Mann–Whitney test. (*, p<0.05).

To further investigate if the differential mRNA expression of MyD88 is associated with similar pattern of regulation at the protein level, we measured the concentration of secreted MyD88 in serum of controls and HCV patients with early and late fibrosis. Similar to the qRT-PCR data, the level of secreted MyD88 from late fibrotic patients was significantly higher than that of early fibrotic patients or healthy controls (Fig 2, p = 0.01 and 0.02, respectively).

## Discussion

Type I IFN orchestrates the expression of various ISGs to subvert virus replication [16] [17]. Our earlier findings demonstrated association between genetic variants of several ISGs and progression of hepatic fibrosis in Egyptian patients infected with HCV genotype 4 [11, 12]. The current data showed that fibrotic HCV patients exhibited transcriptional dysregulation of type I IFN upstream pathway, TLR7. In advanced fibrosis states, expression of TLR7 was diminished, MyD88 and IRF7 was increased whereas NF-κB1 remained unchanged when compared with early fibrosis group or with healthy subjects. The observed tremendous up-regulation of TLR7 related transcripts in F0-F1 patients followed by sharp decline during later stages of fibrosis suggests the importance of this transcript as a potential biomarker for progression of liver disease in HCV infection, a finding that requires further confirmation.

Microarray analysis is widely used to study the impact of HCV on cellular gene profile [9, 18]. We utilized pathway-focused PCR array to examine the transcriptional regulation of type I IFN related genes in HCV-driven liver fibrosis because genome-wide microarray analysis is a hybridization-based technique that is perfect for detecting moderate and highly expressed genes, whereas PCR is an amplification-based method that is perfect for detecting less abundant transcripts [19].

Since HCV is primarily hepatotropic, several studies have focused on analyzing the gene transcription in liver biopsies to identify biomarkers for liver disease progression [8, 18]. However, liver biopsies are highly invasive, and their collections are not easily repeatable, in case follow-ups are recommended. Recent studies showed that viral RNA can be transferred from HCV bearing liver cells to plasmacytoid dendritic cells (pDCs) and induces IFN production [20]. The movement of IFN-producing pDCs back to blood could affect greatly gene expression in PBMCs. The expression of host genes involved in antiviral response was shown to be differentially regulated in PBMCs of chronic HCV patients with different viral RNA concentrations [21] and differential response to IFN treatment [9]. Until now the impact of chronic HCV infection and liver fibrosis on gene expression patterns in peripheral blood cells remains unclear with no evidence of how type I IFN pathway regulates liver fibrosis in genotype 4 infected patients. In the current study, since PBMCs are non invasive research targets and there is a great need to identify therapeutic strategies to revert fibrosis, we attempt to unravel the fact that PBMCs exhibit differential regulation of type I IFN related genes.

In the present investigation, several genes were up regulated in PBMCs derived from advanced fibrotic patients similar to early reports in liver fibrosis induced by HCV or other hepatotropic viruses. Genes encoding IL6, CRP and NOS2 were previously reported to exhibit dysregulation in liver fibrosis. IL6 deficiency was shown to inhibit fibrosis thus indicating its crucial role in fibrogenesis [22, 23]. Also HBV induced liver fibrosis was associated with elevated levels of CRP [24] similar to the current findings in HCV infection. Furthermore, HCV and HBV induced nitric oxide overproduction (that is confined to NOS2) in hepatocytes is involved in progression of chronic viral hepatitis to cirrhosis [25]. The association of other transcripts such as CD70, IFNE and IRF3 with liver fibrosis has been scarcely reported. The observed altered expression of these genes needs to be carried out on larger cohorts of HCV fibrotic patients.

Type III IFNs signaling pathway ends up with the activation of transcription factor complexes ISGF3 (STAT1, STAT2, and IRF-9) which bind to interferon-stimulated response elements in the promoters of several ISGs. The genes induced by type III IFNs are similar to those induced by type I IFN [26, 27]. Our previous work along with other studies did address a strong correlation between the most prominent single nucleotide variation in IFN-λ3 (IL28B rs12979860) and both progression of HCV-induced liver fibrosis [14] and response to IFN treatment [28, 29]. Several studies showed a strong association between IL28B rs12979860 and the expression of hepatic ISGs in case of HCV infection [30, 31]. In our study, the magnitude of gene induction of three ISGs (MX2, IFI6, and IFIT2; which showed altered expression in F2-F4 group compared to F0-F1 group) was not significantly associated with the IL28B rs12979860 genotype. The latter notion suggests that in our patient cohort the induction of these ISGs is primarily under the control of type 1 IFN. These results are in agreement with the previous report which revealed that IL28B rs12979860 genotype does not have impact on ISGs expression in PBMCs from HCV infected patients [32]. In the context of ISGs, we previously showed an association between SNP in MX1-88 and progression of HCV-induced liver fibrosis [11], the current study confirmed that these genetic variants dramatically impact the mRNA expression of MX1. Regardless of the fibrosis score, TT/GT bearing HCV-infected patients exhibited higher levels of MX1 mRNA in PBMCs than GG bearing patients (Data not shown).

TLR7 is an endosomal pathogen recognition receptor that recognizes ssRNA [33]; it is preferentially expressed on pDCs and B cells. The induction of type I IFN by TLR7 is mediated by the recruitment of MyD88, TRAF6, TRAF3, and the kinases IRAK1 and IKKα, and ends up with the activation of IRF7 and NF-**κ**B [34, 35]. HCV is ssRNA virus and in turn can be sensed by TLR7, however little is known about how TLR7 functions in HCV infection and many reports showed contraversal data in this regard. Dolganiuc et al [36] found elevated TLR7 mRNA levels in monocytes and lymphocytes of HCV chronic infected patients compared to controls, indicating the implication of TLR7 in the pathogenesis of HCV infection. Alternately, Iranian [37] and Egyptian patients [38] chronically infected with HCV showed transcriptional downregulation of TLR7 in their blood cells. In addition, human hepatoma cells expressed low levels of TLR7 mRNA following infection with HCV [39]. In our study, we noticed a dramatic increase in TLR7 mRNA level in PBMCs derived from HCV infected patients with early fibrosis followed by attenuation of TLR7 expression in patients with late fibrosis. The elevated levels of TLR7 in early stage of fibrosis is evidence for the implication of TLR7 in the immune response against HCV infection whereas the inversion of the expression pattern in advanced fibrosis patients suggests a protective role of TLR7 against liver fibrosis. TLR7 downregulation in late stage of fibrosis is likely a consequent of immune evasion strategy used by the virus, which might account for its success in establishing chronic infection that ends in cirrhosis. In agreement with the latter speculation, several studies suggest protective role for TLR7 during HCV infection. TLR7 agonists have effectively lower viremia in hepatitis C patients, presumably due to the induction of endogenous IFN production [40, 41]. Adminstration of SM360320 (a synthetic TLR7 activator) led to reduction in HCV mRNA and protein levels in Huh-7 cells [42]. TLR7 showed lower expression in PBMCs [43] and monocytes [10] of chronic HCV patients who did not respond to exogenous IFN than in that of responder patients. Conversely, our results using type 4 infections are different from those findings reported on Indian patients infected with HCV genotype 3 who exhibited elevated levels of TLR7 mRNA in cirrhotic patients [44], suggesting differential impact of genotype 3 vs genotype 4 on TLR7 mRNA abundance and the possible contribution of ethnicity to this context.

Immune activation can be a double-edged sword; our results for MyD88 support this statement. Discordant with TLR7 expression pattern, MyD88 mRNA abundance and circulating protein were found to be higher in HCV patients with advanced fibrosis than in those with early fibrosis, which all showed higher MyD88 expression than that of the healthy subjects. Consistent with the latter finding, MyD88 mRNA expression was increased in monocytes of chronic HCV infected patients [36]. In experimental animal models, attenuation of either liver fibrosis or liver inflammation was associated by reduced MyD88 expression in either nonalcoholic steatohepatitis related hepatic fibrosis [45] or chronic alcohol fed rats [46] respectively. Zhang study [47] revealed increased MyD88 mRNA expression in HBV-transgenic mice having non-alcoholic fatty liver disease as well as in HepG2.2.15 cells treated with stearic acid to induce steatosis. In the current study, the contradiction in the expression pattern of MyD88 and TLR7 is presumably due to the master role that MyD88 acts as a common adaptor protein for TLRs and accordingly its expression might be affected by other signal transduction pathways. Although we found about 2 folds upregulation in IRF7 expression of HCV patients with no fibrosis (F0) compared to controls (data not shown), our data did not reveal any differential regulation of IRF7 and NF-κB1 when the comparison was set between early and late fibrosis groups; it is likely that the expression of both of these nuclear factors is regulated at post transcriptional or post-translational levels. Indeed, the latter conclusion is supported by the fact that the pattern of transcriptional gene expression is not always accompanied by similar pattern of protein expression [48, 49]. Therefore, functional studies are warranted to investigate how these transcription factors are regulated in case of HCV-induced hepatic fibrosis.

This study has several limitations that reduce our power to confirm the significance of the altered transcriptional profile of type I IFN related genes (1) the small sample size, (2) lack of confirmatory experiments at the protein level and absence of functional assays, and (3) lack of information about the main regulator beyond the altered transcriptome and whether it is driven by type I IFN or there is possible role for type III IFN in this context.

To our knowledge this is the first Egyptian study to report the correlation between large panel of type I IFN genes and hepatic fibrosis induced by HCV genotype 4. In conclusion, TLR7 and MyD88 are possible candidates as biomarkers for the progression of HCV-induced liver fibrosis and/ or targets for therapeutic intervention to halt worsening of the clinical course of HCV chronic infection in patients who are uneligible or resistant to DAAs treatment.

## Supporting Information

S1 Fig The expression pattern of MX2, IFI6 and IFIT2 in HCV-chronically infected patients with different IL28B rs12979860 genotypes. qRT-PCR was used to quantify the mRNA expression of IFI6 (A), MX2 (B), and IFIT2 (C) in PBMCs of HCV infected patients with different grades of hepatic fibrosis. The samples were genotyped for IL28B rs12979860 (CC, n = 4 and CT/TT, n = 5). Statistical comparison was performed using Mann–Whitney test.
(TIF)

S1 Table Differential gene expression of type I IFN pathway genes in PBMCs of HCV- chronically infected patients compared to control subjects. (DOCX)
