# Supplementary figures and images for: Transcriptional Dysregulation of Upstream Signaling of IFN Pathway in Chronic HCV Type 4 Induced Liver Fibrosis
Source: PLoS One. 2016 May 2;11(5):e0154512. doi: 10.1371/journal.pone.0154512 (PMC4852926; doi:10.1371/journal.pone.0154512)

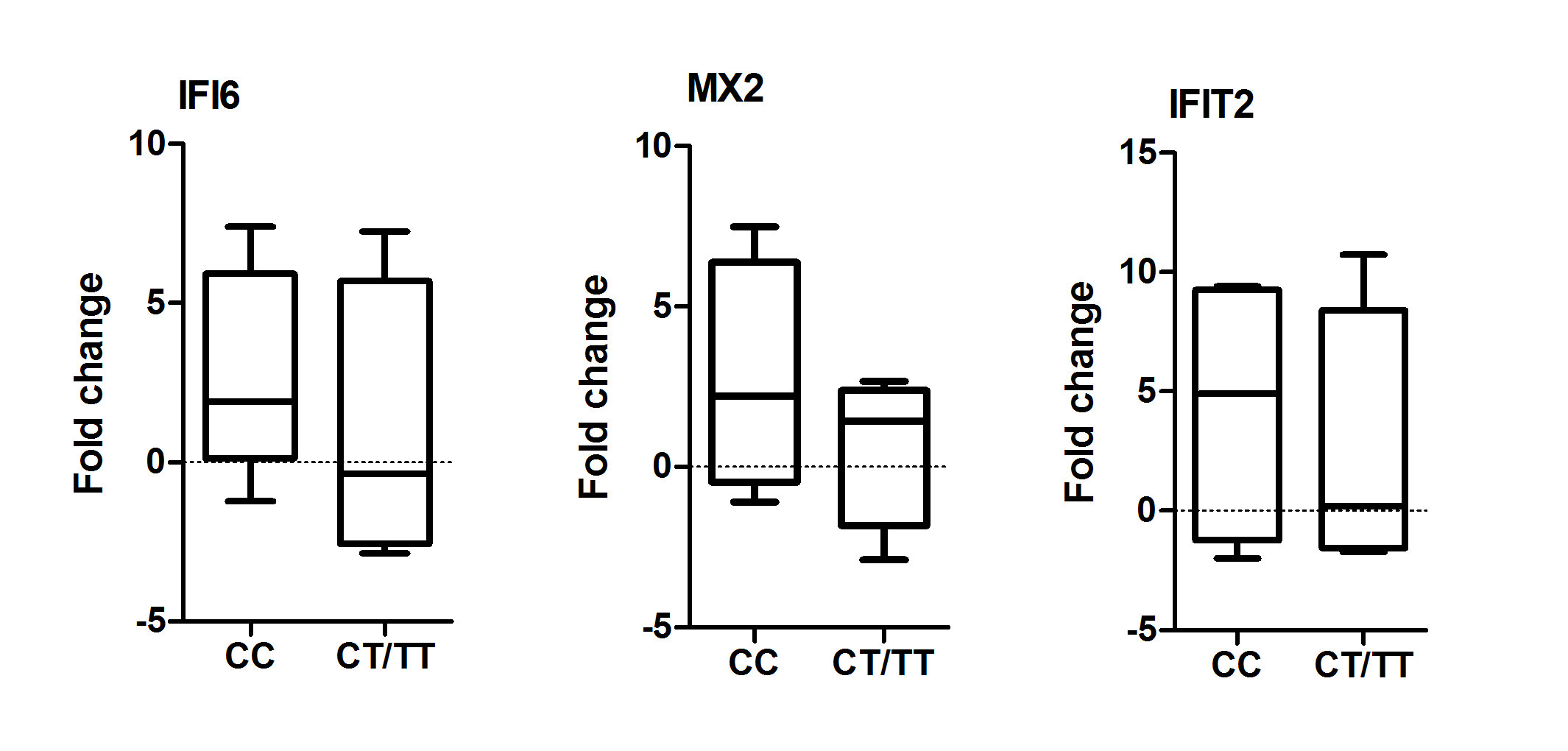

Supplement: S1 Fig — qRT-PCR was used to quantify the mRNA expression of IFI6 (A), MX2 (B), and IFIT2 (C) in PBMCs of HCV infected patients with different grades of hepatic fibrosis. The samples were genotyped for IL28B rs12979860 (CC, n = 4 and CT/TT, n = 5). Statistical comparison was performed using Mann–Whitney test. (TIF) [file pone.0154512.s001.tif]
