# Supplementary material for: Transcriptional Dysregulation of Upstream Signaling of IFN Pathway in Chronic HCV Type 4 Induced Liver Fibrosis
Source: PLoS One. 2016 May 2;11(5):e0154512. doi: 10.1371/journal.pone.0154512 (PMC4852926; doi:10.1371/journal.pone.0154512)
Supplement: S1 Table — (DOCX) [file pone.0154512.s002.docx]

S1 Table. Differential gene expression of type I IFN pathway genes in PBMCs of HCV- chronically infected patients compared to control subjects.

| Gene ID | ^1^Differential expression (F0-F1 /C) | ^1^Differential expression (F2-F4 /C) | P value | Gene ID | ^1^Differential expression (F0-F1 /C) | ^1^Differential expression (F2-F4 /C) | P value |
| --- | --- | --- | --- | --- | --- | --- | --- |
| JAK1 | 2.385 | -11.9021 | 0.001364 | IFNA4 | 10.5854 | 10.5435 | 0.25782 |
| IFIT2 | 4.1756 | -14.7113 | 0.008687 | IFNAR2 | 1.7876 | -1.5293 | 0.258116 |
| TLR7 | 2.0392 | -3.8151 | 0.009661 | IFNA1 | 35.6542 | 30.1005 | 0.260509 |
| STAT2 | 3.8053 | -1.7439 | 0.012764 | IFNW1 | 14.5404 | 19.3772 | 0.264222 |
| MX2 | 2.6354 | -4.0994 | 0.022168 | IRF9 | 3.2043 | 1.593 | 0.283737 |
| IFI6 | 2.8759 | -1.2878 | 0.024341 | IRF1 | 3.0147 | 1.267 | 0.290277 |
| IFNAR1 | 1.8712 | -11.2913 | 0.030398 | EIF2AK2 | 3.0022 | -2.2012 | 0.29421 |
| TICAM1 | 2.2815 | 1.0658 | 0.031933 | IFNE | 14.1037 | 17.9796 | 0.299693 |
| CCL2 | 3.258 | -1.0028 | 0.038246 | CRP | 11.7778 | 25.6647 | 0.317546 |
| IRF7 | 3.595 | -1.5418 | 0.040789 | CD70 | 7.1305 | 4.7016 | 0.324551 |
| TLR8 | -1.71 | -4.4137 | 0.046765 | IFIH1 | -1.0381 | -1.7073 | 0.368298 |
| STAT3 | 3.5259 | -6.7192 | 0.049591 | TRAF3 | 3.5554 | 1.9507 | 0.375562 |
| GBP1 | 16.1113 | 2.526 | 0.056912 | MNDA | 1.9561 | 1.3644 | 0.391554 |
| BAG3 | 2.7132 | -2.1942 | 0.05944 | CIITA | 3.3404 | -1.3947 | 0.407395 |
| IRF2 | 16.5872 | 4.6194 | 0.078215 | JAK2 | 4.7502 | 1.4215 | 0.424128 |
| OAS1 | 2.9567 | -1.6613 | 0.078459 | OAS2 | 2.1347 | -1.165 | 0.424259 |
| IFI27 | 11.6318 | 2.395 | 0.078508 | IL15 | -2.0111 | 1.5305 | 0.425794 |
| IFIT3 | 1.2108 | -8.763 | 0.07954 | ADAR | -1.6911 | -2.213 | 0.431702 |
| MX1 | 1.9697 | -2.1338 | 0.089733 | IFNA2 | 14.3801 | 32.7891 | 0.440384 |
| PRKCZ | 3.5357 | -2.3983 | 0.092733 | CAV1 | 4.9451 | 10.8378 | 0.44483 |
| STAT1 | 3.4295 | -1.1311 | 0.093966 | HLA-B | 876.7368 | 78.7308 | 0.447885 |
| TNFSF10 | 2.5036 | -3.8196 | 0.09781 | VEGFA | 1.7195 | -1.2736 | 0.479532 |
| TLR3 | 3.521 | -1.0852 | 0.106988 | HLA-E | 21.0537 | 14.8049 | 0.509625 |
| TMEM173 | 4.9314 | -1.9019 | 0.123974 | TAP1 | 1.1392 | -1.4053 | 0.531596 |
| SHB | 17.2198 | 4.035 | 0.13446 | CD80 | 9.4742 | 7.2573 | 0.548254 |
| NOS2 | 56.7284 | 20.2722 | 0.136764 | HLA-A | 1.3435 | 1.0771 | 0.555256 |
| PML | 5.3147 | -1.319 | 0.143852 | IRF3 | 3.42 | 3.0902 | 0.564247 |
| IFI16 | 1.7195 | -1.9319 | 0.145477 | TYK2 | 1.136 | -3.0671 | 0.576245 |
| IRF5 | 2.042 | -1.0435 | 0.150911 | SH2D1A | 1.5889 | -1.9453 | 0.600429 |
| IL10 | 15.8895 | 6.5419 | 0.162169 | NMI | 1.8817 | 1.8158 | 0.721959 |
| TLR9 | 2.916 | -1.0062 | 0.163609 | TIMP1 | 3.4011 | 2.7127 | 0.745136 |
| BST2 | 2.3134 | -5.558 | 0.170734 | CASP1 | 1.5094 | -2.762 | 0.756562 |
| PSME2 | 3.4678 | -1.7161 | 0.170914 | MET | 24.2851 | 28.8286 | 0.772253 |
| IFNB1 | 21.3476 | 5.4555 | 0.172887 | MYD88 | 2.1406 | 2.6987 | 0.782765 |
| MAL | 1.5094 | 1.8687 | 0.190336 | CDKN1B | 1.7267 | 1.165 | 0.784827 |
| IFIT1 | 1.539 | -3.6438 | 0.212124 | IL6 | 3.5455 | 4.754 4.754 | 0.804807 |
| IFITM3 | 5.4039 | -2.0123 | 0.214338 | ISG20 | 7.5266 | 6.4839 | 0.86017 |
| SOCS1 | 5.649 | 1.2147 | 0.216849 | CD86 | 1.3623 | 1.3042 | 0.876868 |
| ISG15 | 1.0807 | 1.4226 | 0.229628 | IFITM2 | 3.6859 | 2.5215 | 0.908382 |
| CXCL10 | 1.238 | -3.1652 | 0.242417 | CCL5 | 2.2377 | -1.344 | 0.953087 |
| HLA-G | 5.3889 | -1.1002 | 0.242855 | DDX58 | 1.554 | -1.7791 | 0.95374 |
| IFITM1 | 2.0705 | 3.337 | 0.245975 | IFI30 | 1.9807 | -1.126 | 0.987598 |

**^1^**Differential gene expression was estimated by calculating the ratio of expression of individual gene in the PBMCs from early fibrosis (F0-F1, n=5) and late fibrosis (F2-F4, n=7) HCV-infected patients relative to healthy controls (C, n=5).

A negative sign indicates reduced expression in the patients relative to the normal ones**.**

P value was calculated based on the statistical comparison between F0-F1 and F2-F4 patients.

Genes are arranged in a descending order based on their significant p value.
